# Targeting Metabolic Consequences of Insulin Resistance in Polycystic Ovary Syndrome by D-chiro-inositol and Emerging Nutraceuticals: A Focused Review

**DOI:** 10.3390/jcm9040987

**Published:** 2020-04-02

**Authors:** Sergio Davinelli, Daria Nicolosi, Cinzia Di Cesare, Giovanni Scapagnini, Roberto Di Marco

**Affiliations:** 1 Department of Medicine and Health Sciences “V. Tiberio”, University of Molise, 86100 Campobasso, Italy; cdicesare@unimol.it (C.D.C.); giovanni.scapagnini@unimol.it (G.S.); roberto.dimarco@unimol.it (R.D.M.); 2 Department of Biomedical and Biotechnological Sciences, University of Catania, 95123 Catania, Italy; dnicolosi@unict.it

**Keywords:** polycystic ovary syndrome, d-chiro-inositol, nutraceuticals, insulin resistance, diabetes

## Abstract

Polycystic ovary syndrome (PCOS) is a complex disorder associated with ovarian dysfunction, infertility, menstrual irregularity, and hormonal impairments. Over the last decade, several studies have shown that some PCOS women have insulin resistance (InsR) and hyperinsulinemia, apart from being overweight or obese. Therefore, a crucial clinical aspect is that PCOS patients might develop glucose intolerance and type 2 diabetes. Insulin-sensitizing drugs have been used as first-line treatment to improve hyperinsulinemia in women with PCOS. Although reducing PCOS symptoms and signs, several used insulin-sensitizer drugs may induce side effects, which reduces compliance. D-chiro-inositol (DCI), which is a naturally occurring stereoisomer of inositol, has been classified as an insulin-sensitizer and seems to mitigate multiple InsR-related metabolic alterations in PCOS with a safe profile. However, according to a multi-targeted design, the supplementation with DCI can be synergistically integrated by combining other potential insulin-sensitizing drugs and/or nutraceuticals. The literature provides the initial support for using several unexplored nutraceutical interventions that may target relevant metabolic abnormalities associated with InsR in PCOS. With a need to promote interest in clinical research, this review aims to discuss the efficacy of DCI and the role of emerging nutraceuticals for managing InsR in PCOS.

## 1. Introduction 

Polycystic ovary syndrome (PCOS) is one of the most prevalent endocrine disorders in women of reproductive age and represents one of the leading causes of infertility [1]. PCOS is characterized by different signs and symptoms, and the current diagnosis requires at least two of the following features: anovulation, hyperandrogenism, and ovaries characterized by multiple small follicles at ultrasound examination [2]. However, it has recently been recognized that dysmetabolic features are crucial clinical aspects that need to be considered. As indicated by epidemiological studies, insulin resistance (InsR) and compensatory hyperinsulinemia are present in approximately 80% of obese women with PCOS, and in 30–40% of lean women [3,4]. Clinical manifestations commonly associated with InsR and present in PCOS are hyperandrogenism, menstrual irregularities, and other cardiometabolic complications. Moreover, InsR plays a key role in the pathophysiology of metabolic syndrome (MetS), which increases the risk of developing type 2 diabetes (T2D), cardiovascular disease (CVD), hypertension, and dyslipidemia [5]. Hyperinsulinemia and InsR induce ovarian androgen synthesis and reduce serum sex hormone-binding globulin (SHBG) concentrations, which leads to increased levels of free testosterone. The association between InsR and ovarian hyperandrogenism indicates that insulin influences ovarian function [6]. The first-line treatment of PCOS is lifestyle modification, which is most notably diet. PCOS women, who are not overweight, are often affected by InsR, and modest weight loss improves clinical outcomes in patients of a near-normal body weight [7]. 

However, there is no clear recommendation about the composition of the diet for PCOS women, but the reduction in daily caloric intake, adherence to low-glycaemic index (GI) dietary patterns, and consumption of nutraceuticals derived from food sources may improve insulin sensitivity and metabolic parameters [8,9,10]. Numerous pharmacological and non-pharmacological approaches have been proposed to improve InsR-related metabolic alterations in PCOS. In this context, insulin-sensitizing drugs (e.g., metformin) have been widely used to treat insulin-resistant PCOS patients. These drugs were proven to be effective not only on anovulatory cycles, menstrual irregularity, and hyperandrogenism but also on InsR parameters [11]. However, the cost-effectiveness of this pharmacological treatment, together with patient-related side effects, may reduce subject compliance and limit the use of these drugs [12]. The most common side effects of insulin-sensitizing drugs are nausea, vomiting, abdominal pain, and diarrhea. Recently, oral supplementation with natural substances, such as D-chiro-inositol (DCI), which is a stereoisomer of inositol, have been found to have good efficacy in reducing InsR, while also improving metabolic indices and ovarian function in PCOS patients with fewer side effects [13]. The interest in nutraceutical compounds is growing since they can potentially play a considerable role in managing those patients for whom a pharmacological treatment is still not advisable. The article aims to summarize the current knowledge on the effects of DCI and other unexplored insulin-sensitizing nutraceuticals that may benefit InsR-related conditions in PCOS. 

## 2. Metabolic Consequences of InsR in PCOS

**Figure 1:**
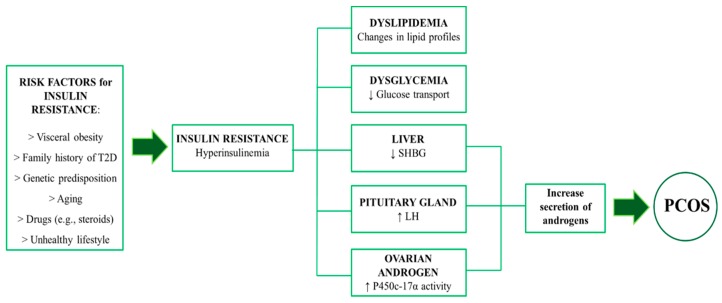
Overview of insulin resistance in PCOS. LH = luteinising hormone. PCOS = polycystic ovary syndrome. SHBG = sex hormone binding globulin. T2D = type-2 diabetes.

Several mechanisms have been proposed for the development of InsR in PCOS. A schematic overview of InsR and its role in the pathogenesis of PCOS is shown in Figure 1. As a compensatory response to InsR, hyperinsulinemia amplifies the effect of the luteinizing hormone (LH) within ovarian theca cells. Resultant activation of P450c17α, which is a key enzyme involved in androgen synthesis, enhances the production of androgens [14,15]. Insulin also increases the adrenocorticotropic hormone (ACTH), which, in turn, mediates adrenal androgen production and accentuates LH-stimulated ovarian steroidogenesis. Moreover, hyperinsulinemia is thought to contribute to hyperandrogenism by inhibiting liver synthesis of SHGB that increases testosterone availability. Current data also suggest that insulin activates the synthesis of androgens in theca cells via multiple pathways, such as the phosphoinositide-3 kinase (PI3K) signalling or the mitogen-activated protein kinase (MAPK) pathway [16]. An additional mechanism is related to abnormal serine and tyrosine phosphorylation of the insulin receptor substrate-1 (IRS-1), which affects metabolic pathways both in classic insulin targets (skeletal muscles and adipocytes) and ovaries [14,17]. Hyperinsulinaemia further exacerbates the pathogenesis of PCOS by inhibiting the production of the insulin-like growth factor-1 (IGF-1) binding protein in the liver, which leads to elevated circulating levels of IGF-1. This, in turn, stimulates ovarian thecal cell androgen production [13,14]. Decreased glucose transporter 4 (GLUT4) in adipocytes, which leads to reduced glucose uptake, is also a contributor to the development of InsR in PCOS women [18]. In combination, hyperinsulinaemia and hyperandrogenaemia can disrupt follicle growth. This is accompanied by menstrual irregularity, anovulatory sub-fertility, and accumulation of immature follicles. A well-written review by Diamanti-Kandarakis describes the modulating action of insulin on ovarian steroidogenesis as well as the importance of the insulin signalling pathway in the control of ovulation [16]. A higher body mass index (BMI) exacerbates InsR in PCOS women more than among those without PCOS [19,20]. Hyperandrogenism promotes android fat distribution in the upper body, both in lean and obese women, when compared with non-PCOS women [21,22]. Androgens seem to play a crucial role in the pathogenesis of metabolic syndrome (MetS) in PCOS subjects. MetS include a cluster of metabolic abnormalities such as obesity, hypertension, and dyslipidemia, and this condition is related to InsR. The prevalence of MetS is higher in hyperandrogenic subjects than non-hyperandrogenic anovulatory women affected by PCOS [23]. The incidence of glucose intolerance and T2D in PCOS patients is 23–35% and 4–10%, respectively, depending on certain risk factors. In lean PCOS women, the incidence is 10–15% and 1–2%, respectively [24,25]. A meta-analysis showed that PCOS women are at a two-fold and a three-fold higher risk of being overweight or obese, compared with non-PCOS individuals. Importantly, this prevalence is affected by ethnicity. Racial and ethnic differences seem to modify the metabolic phenotype of PCOS when compared to InsR [26,27]. Lipid profile abnormalities have also been reported in PCOS women [28]. Dyslipidemia in PCOS women is characterized by high serum triglycerides (TG) and free fatty acid (FFA) concentrations, increased levels of low-density lipoprotein cholesterol (LDL-c), and decreased high-density lipoprotein cholesterol (HDL-c) levels [29]. Another metabolic consequence in PCOS includes hypertension. PCOS women appear to be at increased risk for hypertension. The prevalence of PCOS premenopausal women is estimated at between 9% and 25%, which is higher than the general population [30,31]. 

## 3. The Relevance of D-chiro-inositol and other Nutraceuticals in Insulin Signaling and InsR: Preclinical Evidence

As the relationships between InsR and PCOS manifestations were established over the last decade, the efficacy of insulin-sensitizing compounds has been studied in many experimental conditions [32,33]. DCI was proven to be effective not only on InsR but also on specific clinical outcomes associated with PCOS (e.g., menstrual irregularity, and anovulatory cycles) [34,35]. Various nutraceutical interventions have been investigated in experimental studies, which was followed by clinical trials. PCOS and InsR share several pathophysiological factors. Therefore, it is not unlikely that other natural molecules with insulin-sensitizing activity may improve the sensitivity to insulin in PCOS. For example, *Ipomea Batatas*, *Lagerstroemia Speciosa* (banaba), and lignans extracted from flaxseed (*Linum usitatissiumum*) may be considered as insulin-sensitizers and seem to improve several InsR-related metabolic alterations with a safe pharmacological/nutraceutical profile. 

### 3.1. D-Chiro-Inositol

Inositol and its stereoisomers are considered to be insulin sensitizers and act as mediators of insulin action. The physiological functions of insulin are regulated by two inositol phosphoglycan (IPG) mediators, containing either myo-inositol (MI) or DCI [36]. DCI is synthesized by an epimerase, which is an insulin-dependent enzyme that converts MI into DCI. When InsR occurs, the conversion rate is compromised, reducing the level of DCI in cells. Moreover, it has been demonstrated that the urinary excretion of DCI is reduced individuals affected by T2D [37]. Therefore, the exogenous administration of DCI ensures adequate tissue content, which enhances the activity of the insulin-receptor and reduces glucose levels [38]. Accumulating evidence suggests that DCI improves several cellular events associated with InsR. The PI3K/protein kinase B (Akt) pathway is one of the most important signalling pathways involved in InsR. DCI improves InsR modulating the PI3K/Akt pathway and reducing the concentration of blood glucose in T2D rats. DCI also enhanced the GLUT4 expression on skeletal muscle [39]. Additionally, DCI supports the enhancement of glucose conversion to ATP by increasing its transport in the Krebs cycle. This is achieved by the stimulation of the pyruvate dehydrogenase (PDH) enzyme [40]. Early studies also demonstrated that the administration of DCI improves insulin sensitivity in rhesus monkeys, which enhances insulin action on muscle glycogen synthase and glycogen phosphorylase [41]. Along with these findings, supplementation with DCI reduces InsR in numerous experimental models affected by hyperglycemia or T2D, which also indicates that the DCI may be more effective than MI in restoring insulin sensitivity and glycogen synthesis. However, the current status of the functional role of DCI in insulin action, and its deficit as related to InsR, has been extensively reviewed [42]. 

### 3.2. Flaxseed Lignans

Flaxseed is an important source of several bioactive compounds, including α-linolenic acid (ALA) and lignans. Recently, there has been a growing interest in lignans, a class of phytoestrogens, because of their favourable effects on human health. Flaxseed is considered to be a functional food due to its potential beneficial effects and valuable source of nutrients. Lignans are the major components of flaxseed that have been identified as exhibiting health benefits on metabolic disorders associated with PCOS. Flaxseed lignans are nonsteroidal phytoestrogens that have a chemical structure resembling mammalian estrogens and, hence, it produces estrogen-like effects in mammals. Moreover, flaxseed lignans are metabolized by intestinal bacteria to become bioavailable in the plasma [43]. Lignans derived from flaxseed have gained growing attention due to their multiple biological activities. Flaxseed lignans were reported to lower plasma cholesterol, blood pressure, and glucose concentrations. Additionally, the antioxidant and anti-inflammatory properties of flaxseed lignans have been confirmed in rat models of diabetes and in carbon tetrachloride-induced oxidative stress in rats [44,45,46]. However, although the biological mechanism of lignans on insulin metabolism is not fully elucidated, the antioxidant and anti-inflammatory activities have been considered to contribute to the anti-diabetic benefits of lignans. Fukumitsu et al. investigated the effect of flaxseed lignan metabolites on the development of diet-induced obesity in mice, showing that a high-fat diet (HFD) supplemented with lignans reduced visceral fat, decreased serum insulin, and lowered total cholesterol concentrations. Moreover, administration of flaxseed lignans induced the expression of adipogenesis-related genes, including adiponectin, leptin, GLUT4, and peroxisome proliferator-activated receptor gamma (PPAR-γ) [47]. These effects may improve obesity and reduce the risk of lifestyle-related diseases, including diabetes and hypertension. Wang et al. also showed that the administration of flaxseed lignans to HFD mice lowered fasting blood insulin and FFA levels, and improved insulin tolerance and homeostasis model assessment of insulin resistance (HOMA-IR) by upregulating GLUT4 expression [48]. Recent findings indicate that flaxseed lignans alleviate hepatic steatosis and insulin resistance by enhancing insulin signalling and AMP-activated protein kinase (AMPK) activation [49]. 

### 3.3. Ipomea Batatas

*Ipomoea batatas* (L.) Lam, which is also known as sweet potato, has been used for many years in folk medicine in various parts of the world. For example, the leaves are used to treat T2D by Akan tribes of Ghana [50]. White-skinned sweet potato (WSSP) has also been used in Shikoku, Japan, as a folk medicine to treat diabetes and other diseases. The major phytochemicals present in the leaves of sweet potato are triterpenes, alkaloids, coumarins, flavonoids, saponins, tannins, and phenolic acids [51]. The polyphenols present in the leaves showed various biological functions including radical scavenging properties and antidiabetic activity in vitro and in vivo, which may promote human health. Several investigators found that several phytochemicals from sweet potato had glucose-lowering effects in diabetic animal models. It has been demonstrated that powdered WSSP can improve symptoms of diabetes and decrease high insulin concentrations in streptozotocin-induced diabetic rats and obese Zuker fatty rats [52,53]. Furthermore, oral administration of WSSP to obese Zucker rats for longer than six weeks was shown to reduce the symptoms of hyperinsulinemia and hyperlipidemia by decreasing blood TG and FFA concentrations [53]. More recent in vivo antidiabetic studies have shown that polyphenols such as flavones and caffeoylquinic acid modulate lipid metabolism. This improves blood glucose concentration and reduces the incidence of diabetic complications caused by blood lipid abnormalities and insR [54,55]. 

### 3.4. Lagerstroemia Speciosa (banaba)

The first animal study on the glucose-lowering effects of *Lagerstroemia Speciosa* (banaba) was reported by Garcia in 1940 [56]. Several experimental studies in animal models have subsequently shown that banaba extracts exert beneficial effects on blood glucose and lipid regulation. The glucose-lowering effects of banaba have been attributed to corosolic, acid, and ellagitannins. The biological action of these compounds on glucose and lipid metabolism involves multiple mechanisms, such as enhanced cellular uptake of glucose, decreased gluconeogenesis, and regulation of lipid metabolism. These effects may be mediated by PPAR, MAPK, and nuclear factor kappa-B (NF-kB) [57]. Leaf banaba extracts effectively control hyperglycemia and hyperinsulinemia by reducing blood glucose, insulin, TG, and glycated haemoglobin levels in diabetic mice. Furthermore, these effects were associated with increased expressions of liver PPAR-α mRNA and adipose tissue PPAR-γ mRNA. These results suggest that the banaba extract may improve insulin sensitivity by regulating PPAR-mediated lipid metabolism [58]. Three ellagitannins extracted from banaba have also shown to increase glucose uptake in isolated rat adipocytes [59]. Miura et al. conducted numerous studies on diabetic mice treated with corosolic acid. This compound, at a single dose of 10 mg/kg, significantly reduced blood sugar levels, which increased the expression of GLUT4. The same authors showed that even a single dose of 2 mg/kg corosolic acid may reduce blood sugar levels. This supports the hypothesis that corosolic acid improves glucose metabolism by reducing InsR [60,61]. An interesting study examined the effects of corosolic acid on osteoblastic bone formation. The effects appear to be mediated by the modulation of MAPK and NF-kB [62]. Recent findings also suggest that corosolic acid protects renal damage in diabetic animals. This compound inhibits the proliferation of diabetic glomerular mesangial cells via MAPK signalling pathways [63].

## 4. Clinical Potential for improving InsR in PCOS

Numerous nutraceutical-based treatments have been investigated in women with PCOS to target the metabolic outcomes associated with this condition (Table 1). The effects of oral DCI supplementation in women with PCOS have been evaluated in several intervention trials with a daily dosage ranging from 500 to 1200 mg, over a period from 6 to 24 weeks. A randomized, double-blind controlled trial was conducted to determine whether oral DCI modulates an increase in the release of the DCI-IPG mediator and an improvement in insulin sensitivity in women with PCOS. After six weeks of DCI supplementation, it has been shown that increased release of DCI-IPG was significantly associated with improved insulin sensitivity. These findings suggest that the DCI-IPG mediator may be a target for therapeutic interventions in PCOS [64]. The oral administration of 1200 mg of DCI once daily for six to eight weeks in 44 obese women with the PCOS improved ovulatory function and decreased serum androgen concentrations, blood pressure, and plasma TG concentrations [65]. Genazzani et al. observed that DCI administration positively affect insulin sensitivity in obese PCOS patients, revealing that supplementation with DCI (500 mg/day for 12 weeks) is effective in those PCOS patients who have a family history of T2D [66]. PCOS has been associated with increased generation of reactive oxygen species (ROS). The resultant oxidative stress induces a pro-inflammatory state that may contribute to InsR and hyperandrogenism in PCOS [67]. Clinical evidence suggests that treatment with oral DCI 1000 mg daily decreases the production of ROS in ovarian follicular fluid obtained from women with PCOS [68]. Although DCI has been shown to be effective against InsR, other integrative approaches should be considered to expand the therapeutic armamentarium for women with InsR and PCOS. Moreover, given the frequent concurrence of PCOS and InsR, and the mechanistic similarities between these two conditions, it is crucial to investigate the clinical relevance of other natural molecules with insulin-sensitizing activity. So far, there is a paucity of clinical studies exploring the insulin-sensitizing activities of nutraceuticals in the PCOS setting. However, numerous safe and well-tolerable compounds have emerged as relevant to treat InsR and, therefore, potentially useful to improve the sensitivity to insulin in PCOS. Several randomized clinical trials have been conducted to investigate the efficacy of flaxseed or its derivatives on glycemic control, insulin sensitivity, and lipid metabolism. A recent randomized controlled clinical trial found evidence that flaxseed supplementation in patients with PCOS may improve dyslipidemia, obesity, InsR, and inflammation. In particular, the results indicated that 12 weeks of flaxseed powder supplementation had beneficial effects on insulin metabolism parameters, body composition, high-sensitivity C-reactive protein (hs-CRP), TG, HDL-c, leptin, and hirsutism [69]. A systematic literature search was performed by Pan et al. to assess the effects of flaxseed on lipid abnormalities. Flaxseed or its bioactive compounds significantly reduced total cholesterol and LDL-c concentrations. However, these changes were dependent on the type of intervention, initial lipid concentrations, and characteristics of the subjects (e.g., sex or age) [70]. There are conflicting results regarding the effects of flaxseed on glycemia or insulin sensitivity. A meta-analysis was conducted to sum the data from 25 randomized clinical trials and draw a better conclusion. The results indicate that flaxseed supplementation may improve glycemic control. The changes may be more pronounced with whole flaxseed consumption, in subjects with higher baseline glucose levels, and in interventions longer than twelve weeks [71]. Adiponectin, which is produced by adipocytes and acts as a modulator of insulin sensitivity, has been shown to be low in insulin-resistant states. A multicentre study elucidated the efficacy of *Ipomoea batatas* on circulating adiponectin levels and insulin sensitivity. After five months of treatment, the study has shown an increase in adiponectin associated with the improvement of insulin sensitivity and glycated haemoglobin in patients with T2D [72]. In addition, a recent clinical trial by Shih et al. demonstrated that *Ipomoea batatas* effectively reduces glycated haemoglobin [73]. Several clinical data from small intervention trials reveal that the extracts of banaba exhibit significant glucose-lowering effects in humans. After two weeks of treatment, a dose-dependence study shows that oral formulations of an extract from the leaves of banaba standardized to 1% corosolic acid exert a 30% decrease in blood glucose levels of subjects affected by T2D [74]. In a study conducted by Tsuchibe et al., healthy subjects with a baseline blood glucose level of 104 mg/dL received 10 mg corosolic acid extracted from banaba and standardized to 18% corosolic acid. After two weeks of supplementation, the authors observed a 12% decrease in fasting and postprandial glucose concentrations [75]. Fukushima et al. also clarified the effect of corosolic acid from banaba on post challenge plasma glucose levels in humans. In this study, 31 subjects were orally administered 10 mg corosolic acid in a double-blind and cross-over design. The capsules were given 5 min before a 75 g oral glucose tolerance test. Blood glucose levels were measured at 30 min intervals for 2 h. The authors observed that the treatment lowered blood glucose levels when compared with controls, and the values were statistically significant at the 90-min time point [76]. Overall, these clinical studies demonstrate that DCI and the above-mentioned nutraceuticals, alone and in combination, have potential clinical applications to improve insulin sensitivity and metabolic abnormalities associated with PCOS.

## 5. Conclusions

There has been a continuous growth in the nutraceutical field in recent years, becoming a key research area associated with health improvement and disease prevention [77,78,79]. PCOS is not only a reproductive pathology but also a systemic condition and its etiopathogenesis is still not completely understood. In the past, therapy for PCOS has been foused on treatment of hirsutism and restoration of ovulation. Currently, a major challenge in PCOS pathogenesis research is to clarify the complicated relationship between InsR and the development of PCOS. Recently, the approach of clinical practice has been a progressive changed and improved toward prevention together with standard treatments [80]. Pharmacologic reduction in insulin levels may ameliorate complications associated with hyperinsulinemia and hyperandrogenemia and appear to offer a therapeutic modality for PCOS. Current therapeutic tools are represented by hormonal contraceptives, anti-androgen drugs, and insulin-sensitizing drugs. However, several of these approaches, including the use of insulin-sensitizing drugs, often induce side effects. Therefore, new alternative strategies have been proposed to treat and or prevent InsR and metabolic abnormalities in PCOS [81]. The use of nutraceutical compounds may offer a new avenue for several adjunctive treatment strategies [82,83]. Based on available evidence, DCI may improve metabolic/glycemic abnormalities in PCOS patients. Whether this translates into clinical benefit with a reduced onset of metabolic complications remains to be confirmed. Given the lack of significant adverse effects, DCI is an attractive treatment option to modulate insulin metabolism and orchestrate ovarian function. Moreover, a relatively large number of nutraceuticals have been studied to improve metabolic parameters and InsR in PCOS patients. Although recommendations for clinical use are premature, bioactive compounds from flaxseed, sweet potato, and banaba may target relevant underlying pathways involved in the metabolic dysfunctions associated with PCOS. The results discussed in this case provide preliminary evidence for the use of these nutraceuticals to improve a glycemic profile and cardiometabolic abnormalities in women with PCOS. We have highlighted the possibility that these nutraceticals may potentially expand the armamentarium available to physicians. However, clinical data are still preliminary, and the results need to be replicated in other settings, especially PCOS, and on well-selected populations. Higher powered trials are required to expand limited literature and investigate whether these nutraceuticals may be an adjunctive treatment to target clinical metabolic outcomes associated with PCOS.

## Figures and Tables

**Table 1:** Summary of the cited clinical studies and meta-analyses involving the use of d-chiro-inositol, flaxseed, sweet potato, and banaba.

Author	Intervention	Study Design	Population	Dosage/Duration	Outcomes	Results
Cheang et al. [64]	DCI supplementation	RCT with placebo	11 women (18–40 y) with PCOS	1200 mg twice daily for 6 weeks	Hormones, plasma DCI, DCI-IPG release, insulin sensitivity	Significant relationship between DCI-IPG release and insulin sensitivity
Nestler et al. [65]	DCI supplementation	RCT with placebo	44 obese women (18–40 y) with PCOS	1200 mg/day for 6 to 8 weeks	Hormones, lipid profiles, BP, plasma insulin	Increase of the action of insulin, improvement of ovulatory function and decrease androgens, BP, and TG
Genazzani et al. [66]	DCI supplementation	Intervention trial	22 obese women (age not reported) with PCOS	500 mg/day for 12 weeks	Hormones and plasma insulin	Improvement of hormonal pattern, especially LH and FSH, and restores insulin sensitivity
De Leo et al. [68].	DCI supplementation	Intervention trial	20 women (age not reported) with PCOS	500 mg twice daily for12 weeks	Oxidative stress on follicular fluids	Reduction of the oxidation of thiol groups
Haidari et al. [69]	Flaxseed powder supplementation	Open label RCT	41 patients (18–45 y) with PCOS	30 g/day for 12 weeks	Anthropometric and biochemical parameters	Reduction in body weight, HOMA-IR, TG, hs-CRP and leptin, and increase in QUIKI, and HDL
Pan et al. [70]	Supplementation with flaxseed and its derivatives	Meta-analysis of 28 RCTs	1539 subjects (age not reported) with HC, T2D, or healthy	Median dose 38 g; median duration 8.5 weeks	Blood lipid concentrations	Reduction in total and LDL-cholesterol
Mohammadi-Sartang et al. [71]	Supplementation with whole flaxseed, flaxseed oil, and lignan extract	Meta-analysis of 25 RCTs	2080 subjects (mean age from 29.4 to 67.6 y) with PCOS, HC, T2D, CVD, MetS, obesity, or healthy	Whole flaxseed from 10–60 g ALA from 1 to 15 g; Lignans from 21 to 600 mg Duration from 2 to 48 weeks	Glucose control and insulin sensitivity	Reduction in blood glucose, insulin levels, and increase in QUIKI
Ludvik et al. [72]	Supplementation with sweet potato	RCT with Placebo	61 patients (mean age 57.2 y) with T2D	4 g/day for 20 weeks	Insulin sensitivity, T2D parameters, lipids, adiponectin, hs-CRP, and fibrinogen	Improvement in HbA1c, TG, adiponectin, fibrinogen, and insulin sensitivity
Shih et al. [73]	Supplementation with sweet potato	RCT with no placebo	56 overweight (mean age 38.7 y) participants	132 g/day for 8 weeks	Anthropometric and biochemical parameters	Improvement in HbA1c, and reduction in BMI
Judy et al. [74]	Supplementation with an extract from banaba	RCT with no placebo	10 subjects (55–70 y) With T2D	16, 32 and 48 mg/day for 2 weeks	Blood glucose levels	Reduction in blood glucose levels
Tsuchibe et al. [75]	Supplementation with corosolic acid extracted from banaba	RCT with no placebo	12 healthy subjects (mean age 57.7 y)	10 mg/day for 2 weeks	Postprandial blood glucose and anthropometric parameters	Inhibitory effect on postprandial blood glucose. Reduction in BMI.
Fukushima et al. [76]	Supplementation with corosolic acid extracted from banaba	double-blind and cross-over RCT	31 subjects (mean age 51.6 y) with T2D and impaired glucose tolerance	10 mg; different time points	Fasting plasma glucose	Lowering effect on post-challenge plasma glucose levels

Abbreviation: D-chiro-inositol, DCI. Randomized controlled trial, RCT. Polycystic ovary syndrome, PCOS. Inositolphosphoglycan, IPG. Blood pressure, BP. Triglycerides, TG. Luteinizing hormone, LH. Follicle-stimulating hormone, FSH. Homeostatic model assessment of insulin resistance, HOMA-IR. High-sensitivity C-Reactive Protein, hs-CRP. Quantitative Insulin-Sensitivity Check Index, QUIKI. High Density Lipoprotein, HDL. Hypercholesterolemia, HC. Type 2 diabetes, T2D. Low-density lipoprotein, LDL. Cardiovascular disease, CVD. Metabolic syndrome, MetS. a-linolenic acid, ALA. Glycated haemoglobin, HbA1c. Body mass index, BMI.
